# Self-Perceived Life Satisfaction during the First Wave of the COVID-19 Pandemic in Sweden: A Cross-Sectional Study

**DOI:** 10.3390/ijerph18126234

**Published:** 2021-06-09

**Authors:** Christina Brogårdh, Catharina Sjödahl Hammarlund, Frida Eek, Kjerstin Stigmar, Ingrid Lindgren, Anna Trulsson Schouenborg, Eva Ekvall Hansson

**Affiliations:** 1 Department of Health Sciences, Lund University, 221 00 Lund, Sweden; catharina.sjodahl_hammarlund@med.lu.se (C.S.H.); frida.eek@med.lu.se (F.E.); kjerstin.stigmar@med.lu.se (K.S.); Ingrid.lindgren@med.lu.se (I.L.); anna.trulsson_schouenborg@med.lu.se (A.T.S.); eva.ekvall_hansson@med.lu.se (E.E.H.); 2 Department of Neurology, Rehabilitation Medicine, Memory Disorders and Geriatrics, Skåne University Hospital, 222 41 Lund, Sweden; 3 The PRO-CARE Group, School of Health and Society, Kristianstad University, 291 39 Kristianstad, Sweden; 4 Department of Research and Development, Skåne University Hospital, 222 41 Lund, Sweden; 5 Department of Neurosurgery and Pain Rehabilitation, Skåne University Hospital, 222 41 Lund, Sweden

**Keywords:** life satisfaction, COVID-19 pandemic, health

## Abstract

Currently, there is limited knowledge on how the Swedish strategy with more lenient public health restrictions during the COVID-19 pandemic has influenced people’s life satisfaction. Here, we investigated self-reported life satisfaction during the first wave of the pandemic in Sweden, and perceived changes in life satisfaction in relation to various sociodemographic factors. A total of 1082 people (mean age 48 (SD 12.2); 82% women) responded to an online survey during autumn 2020 including the “Life Satisfaction Questionnaire-11”. A majority (69%) were satisfied with life as a whole, and with other important life domains, with the exception of contact with friends and sexual life. An equal share reported that life as a whole had either deteriorated (28%) or improved (29%). Of those that perceived a deterioration, 95% considered it to be due to the pandemic. Regarding deteriorated satisfaction with life as a whole, higher odds were found in the following groups: having no children living at home; being middle aged; having other sources of income than being employed; and having a chronic disease. The Swedish strategy might have contributed to the high proportion of satisfied people. Those who perceived a deterioration in life satisfaction may, however, need attention from Swedish Welfare Authorities.

## 1. Introduction

In March 2020, the World Health Organization declared COVID-19 to be a public health emergency of international concern [1,2]. Even though the symptoms of COVID-19 may be asymptomatic or mild for some, others will develop pneumonia and serious life-threatening complications. In particular, COVID-19 could be dangerous for older people and people with underlying chronic diseases [3,4]. In order to reduce the rapid spread of COVID-19, and its potential harmful effects for physical health, governments have taken several actions worldwide. Many countries have imposed lockdowns and people have been in quarantine in order to mitigate the impact of the virus and flatten the rising curve of cases and deaths [1,5,6].

Although the lockdowns, for example in Europe, the United States, Asia and the Middle East have been effective in preventing the spread of COVID-19 [5,7], everyday life has significantly changed for numerous people [8,9,10]. The restrictions and home confinements following the pandemic limited social activities and participation, leading to various levels of physical, psychological and psychosocial distress [8,11,12], especially for vulnerable people [13]. Additionally, feelings of uncertainty, anxiety, fear of contracting the disease and fear of death have been reported [14]. In particular, belonging to the younger age group, being separated, symptoms of depression, difficulties in regulating emotions and poor sleep quality [15] have been shown to be risk factors for negatively experiencing loneliness during the pandemic.

In Sweden, societal lockdown has not been possible due to current legislation [6,16]. Instead, the country has followed a different path with less rigorous restrictions based on governmental recommendations and individual responsibility. The Public Health Agency of Sweden has recommended physical distancing, frequent and careful handwashing, working from or studying at home when possible, avoiding interaction with people in various social contexts, and staying at home when having the slightest symptoms of infection [6]. People 70 years and older, have been given even stricter recommendations to avoid social contacts and gatherings in order to reduce the risk of getting infected [17]. Visiting relatives and family members in their nursing homes was prohibited in Sweden following the high mortality rates among older people [1,6,18]. However, important infrastructural functions such as transport, schools for children, shops/malls, and gyms have partly been running with the incentive to reduce the socioeconomic consequences as well as the potential effects on physical and mental health [6,16,19].

Given that change can have an impact on physical and mental well-being [5,20,21,22], it is important to study how people’s perceived life satisfaction has changed during the pandemic in Sweden. Previous worldwide studies have shown that restrictions following the pandemic have resulted in reduced social interaction and life satisfaction [5], leading to more distress, depression, anxiety [23,24], and financial worries [11,12]. Thus, even though the Swedish government has applied more lenient public health restrictions, there are reasons to believe that people’s perceived life satisfaction has been affected in terms of various important life domains.

Currently, there is limited knowledge of how the restrictions during the COVID-19 have influenced people’s life satisfaction and well-being in Sweden. In the early stage of the pandemic, on average, older adults in Sweden rated their well-being as high, or even higher, than for the same period the previous year. Those who reported lower well-being were more worried about negative health and socioeconomic consequences of the pandemic [17]. As knowledge is scarce on how the Swedish strategy has influenced peoples’ life satisfaction during the continuation of the pandemic, the aim of this study was to investigate self-reported life satisfaction during the first wave of COVID-19, and perceived changes in life satisfaction compared to the same period of the previous year. We also explored whether perceived changes differed between gender, age groups, family situations, geographical areas, chronic diseases, place of residence, and country of birth. This study was explorative in nature. We expected, however, that people living in Stockholm and Gothenburg—areas that have had larger outbreaks during the first wave of the pandemic and hence stricter restrictions—and people suffering from a chronic disease would have higher odds for perceiving deteriorated life satisfaction.

## 2. Materials and Methods

### 2.1. Recruitment of Participants

To recruit participants to this study, a Facebook announcement from the Department of Health Sciences, Lund University was posted between 1 September and 7 October 2020. The announcement was also spread via Instagram and Twitter and comprised information about this project. The announcement was directed to three different regions of Sweden—two regions with large outbreaks of the pandemic (Stockholm, the capital and Gothenburg, the second largest city), and to the county of Scania in the southernmost part of Sweden. However, the invitation could also be shared by Facebook users to people outside these areas. Individuals, aged 18 years or older, able to read and understand Swedish were invited to participate in this project.

### 2.2. Ethics

On the Facebook page, there was a link to a webpage hosted at Lund University.se, with general information about this project, information on participation and a link directing the participants to the survey. Each participant consented to partake in this study by clicking on the following link (https://rcweb.med.lu.se/surveys/?s=KTYLPACNK3) (accessed on 1 September 2020) and starting the survey. This study was approved by the Swedish Ethical Review Authority (Dno. 2020-02776), and the principles of the Declaration of Helsinki were followed.

### 2.3. Survey and Data Collection

Data were collected using the Research Electronic Data Capture (REDCap) program, which is a means of capturing, processing, and integrating data on a compliant electronic data capture system at Lund University [25,26]. The survey included sociodemographic questions regarding gender, age, marital status, family situation, geographical area, place of residence, country of birth, education, and sources of income. The participants also reported whether they had any ongoing diseases or not, and responded to questions regarding physical activity [21] and perceived life satisfaction according to the Life Satisfaction Questionnaire (LiSat-11) [27,28,29]. In the present study, only data on sociodemographics and perceived life satisfaction (current ratings, and perceived changes) are reported.

### 2.4. Life Satisfaction Questionnaire

LiSat-11 is a generic questionnaire that assesses perceived life satisfaction [21,29,30,31,32,33]. It consists of one global item “life as a whole” and 10 domain-specific items regarding vocation, economy, leisure, contacts with friends and acquaintances, sexual life, activities of daily living (ADL, i.e., ability to manage self-care in dressing, hygiene, transfers), family life, partnership/relationship, physical health and psychological health. Each item is rated as: 1 (very dissatisfying), 2 (dissatisfying), 3 (rather dissatisfying), 4 (rather satisfying), 5 (satisfying) and 6 (very satisfying), and higher scores indicate a greater level of perceived satisfaction. The scores can be dichotomized into two categories—dissatisfied (scores 1 to 4) and satisfied (scores 5 and 6) [29]. The global item “life as a whole” can be used as a single measure of perceived life satisfaction [29]. LiSat-11 was shown to be reliable in various populations [30,31,34], and values from a Swedish reference sample are available [29].

In addition to ratings of perceived life satisfaction, the participants also reported perceived changes in the different domains of LiSat-11 compared to the same period the previous year (i.e., as “deteriorated”, “unchanged” or “improved”). If they perceived a change, they also reported whether they considered the change to be due to the pandemic by answering “yes”, “partly” or “not at all” or “do not know”.

### 2.5. Data Analysis

LiSat scores for each individual item are presented as the mean (SD) and median (IQR), as well as the proportion (%) of participants being satisfied (scores 5 and 6) for each item [29]. The proportion (%) of participants who perceived that different aspects of LiSat-11 had “deteriorated”, were “unchanged” or had “improved” compared to the same period the previous year are presented, along with the proportion of participants who perceived a deteriorated or improved change of LiSat-11 to be due to the pandemic (“yes”, “no” or “partly”). In addition, the proportion of participants who attributed the change in life satisfaction to the pandemic was compared between those that perceived a deterioration or improvement, by use of the chi square test.

Group comparisons regarding changes in life satisfaction were based on the categorical responses of perceived changes of item 1 in LiSat, “life as a whole”. Chi square tests were performed in order to explore whether the proportion of participants reporting “deteriorated”, “unchanged” or “improved” life satisfaction differed between groups based on gender, age, marital status, family situation, geographical area, residential communities, country of birth, education and whether they suffered from an ongoing disease or not. The perceived change in “life as a whole” was further dichotomized into two variables—“deteriorated satisfaction” (vs no change or improvement) and “improved satisfaction” (vs no change or deterioration). Variables where the chi square test indicated a significant difference regarding perceived change (i.e., deterioration, improvement, or no change) between the groups were entered in two separate multivariable logistic regression models with the dichotomized variables “deteriorated” and “improved” satisfaction as outcomes. Odds ratios (OR) with corresponding 95% confidence intervals (95% CI) are presented.

Significance level was set to *p* < 0.05. Statistical analyses were performed in Statistical Package for Social Sciences (SPSS) v 26.

## 3. Results

Of the 1082 participants, a large proportion were women (82%). A majority had graduate degrees from tertiary level education (85%), were married/cohabitating (74%) and aged between 35 and 69 years (81%). Less than half lived in Scania, the southernmost part of Sweden (43%), 37% lived in the two biggest cities in Sweden, and 31% lived in a village. Seventy-nine percent of the participants were employed and 55% had no children living at home. Approximately one-quarter suffered from a chronic disease (Table 1).

The ratings of LiSat-11 showed that a large proportion were satisfied with life as a whole (69%), with their ability to manage activities of daily living (97%), their family life (73%), partnership/relationships (71%) and with their financial situation (70%). A smaller proportion were satisfied with contact with friends (43%) and their sexual life (35%) (Table 2).

For the various LiSat-11 items, 42% to 94% of the participants reported no change as compared to the same period the previous year. Approximately half reported that their contact with friends had deteriorated (52%), and about one-third rated leisure time activities (33%) and life as a whole (28%) as deteriorated. Between 21% and 24% reported a deterioration in vocational situation, as well as in physical and psychological health. Approximately one-third reported an improvement in life as a whole (29%) and a slightly smaller proportion considered that physical health (26%), leisure time activities (24%), the vocational situation (25%) and the financial situation (20%) had improved.

For all items, except sexual life and partner/relationship, the proportion of those who considered that the change was due to the pandemic was significantly larger among those who experienced a deterioration, compared with those who experienced an improvement (*p* ≤ 0.001). For example, regarding life as a whole, contact with friends and leisure time activities more than 90% of those experiencing deterioration considered the deterioration to be due to the pandemic. One exception was sexual life, in which only approximately one-third considered that the deterioration was due to the pandemic (Table 3).

When we compared the proportions of participants reporting changed satisfaction with life as a whole, significant differences were seen between gender, age groups, family situation, geographical areas, place of residence, occupation and chronic disease (*p* ≤ 0.001–0.028) (Table 4). These variables were therefore included in the multivariable logistic regression.

In the final multivariable logistic regression, significantly higher odds for experiencing deteriorated satisfaction with life as a whole were seen in the age group 35–49 compared to those younger than 35 (OR 1.75, 95% CI 1.08–2.85), among people living in Stockholm compared to those living in Scania (OR 1.55, 95% CI 1.00–2.40), for those having no children living at home compared to having children living at home full time (OR 1.83, 95% CI 1.28–2.62), for those having other sources of income than being employed (OR 1.65, 95% CI 1.13–2.06), and for those suffering from a chronic disease (OR 1.50, 95% CI 1.10–2.41). Significantly lower odds for experiencing improved life satisfaction were seen in individuals 35 years and older compared to the youngest age group (<35) and in individuals without children living at home (Table 5).

## 4. Discussion

The main findings in the present study were that a majority of the participants (69%) reported a high level of satisfaction with life as a whole. Likewise, almost all of the other aspects of life satisfaction were perceived as satisfying or very satisfying, with the exception of contact with friends and sexual life. For many items in LiSat-11, the majority reported no changes compared to the same period last year. However, approximately one-third perceived that life as a whole had deteriorated, and an equal share perceived that it had improved. Of those that perceived a deterioration, 95% considered it to be due to the pandemic. Having no children living at home, being middle aged, having other sources of income than being employed, and suffering from a chronic disease were associated with significantly higher odds for experiencing deteriorated satisfaction with life as a whole, compared with reference groups.

More than two-thirds of our participants were satisfied with life as a whole during the first wave of the pandemic. Our finding is in agreement with a Swedish reference sample as described by Fugl-Meyer et al. 2002 [29], where 70% were satisfied with life as a whole. A possible reason for the similar figures could be the relatively lenient public health restrictions that Sweden has applied during the first wave of COVID-19. Moreover, a previous study has shown that the global item life as a whole is significantly associated with most of the other domain-specific items in LiSat-11 [30]. As our participants were satisfied or very satisfied with many items in LiSat-11 except with contact with friends and their sexual life, this may reflect why a high proportion also was satisfied with life as a whole.

Changes in life as a whole differed, however, between the groups in our sample. Younger people (below 35 years of age) reported an improvement in life as a whole, to a higher extent. Our findings are partly in agreement with research from countries that have been locked-down. Ogden et al. reported that younger people who were socially active felt that time passed more quickly during the lockdown [35]. Younger people may have adapted better to the new circumstances following the pandemic, as an increased number of younger people are connected through digital technology [5], which may reduce social distress.

Moreover, less than half (43%) of our participants were satisfied with their contact with friends and only 35% were satisfied with their sexual life. Our figures are somewhat lower than the Swedish reference values [30], where 65% and 56%, respectively were satisfied with these domains. Of those who reported that contact with friends had deteriorated, almost all thought that it was due to the pandemic. Other studies have shown that the hard restrictions in Europe and other parts of the world could cause changes in the life situation and that reduced social contacts are associated with impaired mental health, psychosocial distress, health anxiety and loneliness [5,7,8]. Loneliness, in turn, may arouse anxiety and cause fragmented sleep [4].

Most of our participants were married or cohabiting (74%) and satisfied with their family life (73%) and their partnership/relationships (71%). Having a partner and living in a good relationship is shown to be related to better mental health [9]. Pieh et al. reported that being married was not a factor for improved mental health in itself, but that perceived support and satisfaction in the relationship was most important [9]. Previous studies have also shown that married people adapted better to the restrictions during the pandemic compared to single people [13,36].

A majority of the participants in our study reported no change in their financial situation (69%) or in their vocational situation (51%). However, a deterioration was reported by 11% and 24%, respectively. These findings may be due to the decision of the Swedish government to keep all preschool and elementary schools open for children, which might have had a positive impact on the economy [37]. Although the Swedish people were required to follow recommendations of physical distancing, public and recreational facilities, restaurants, shops, gyms, etc., were still accessible [19], which may explain the results.

An increase in unemployment in Sweden has been registered during the pandemic, even though the Swedish strategy did not include a lockdown [38]. In our sample, 24% reported that their vocational situation had deteriorated, and 87% of those perceived that this change was due to the pandemic. In the studies from Europe, the United States, and China that have been locked-down, it is described that the growing numbers of job losses and unemployment have caused an existential crisis, with the fear of not being able to provide for oneself and one’s family [11,39]. In addition, being at work also contributes to social connection and setting personal goals and brings about a sense of self-determination [40]. People who had to stop working due to home confinement report worse physical and mental health and increased levels of distress [41]. It is also possible that the people in our sample were afraid of losing their jobs and missed socializing with colleagues, and therefore rated their vocational situation as deteriorated, which is in line with previous studies [18,42,43].

Furthermore, 20% of our sample reported that their psychological health had deteriorated, and 90% of those perceived that it was due to the pandemic. Other studies from Europe, Asia, and Africa [22], as well as from China and Canada [44], have reported increased anxiety and depression related to a fear of catching the infection, and psychological and emotional distress [23], already after two weeks of isolation in the early stages of COVID-19. The changes in lifestyle may also affect sleep quality negatively, which has been associated with depression, fatigue and psychological distress [45,46].

Regarding physical health, more than half (52%) of our participants reported unchanged satisfaction during the first wave of the pandemic, whereas approximately one-quarter reported deteriorated or improved satisfaction with physical health. A recently published study from Sweden [21], which included the same population as in the present study, showed that 36% of the participants had increased their physical activity (PA) during the pandemic, whereas 29% had decreased their PA. Those who reported decreased levels of PA perceived significantly lower life satisfaction than the groups with maintained or unchanged activity. Another multicenter study from Europe, Northern Africa, South America Canada, China, and Australia has reported that the confinement following the pandemic resulted in reduced physical activity on all levels due to, for example, curfews and closed gyms in some countries [20]. Sedentary time and prolonged sitting also increased several hours per day [20,47], as well as unhealthy eating behaviors to mitigate anxiety and stress, which may have affected perceived physical health negatively [48].

In the present study, significantly higher odds for experiencing deteriorated satisfaction with life as a whole were found among middle-aged individuals, individuals with no children living at home, living in Stockholm, having other sources of income than being employed, and having a chronic disease, compared with reference groups. This partly confirmed our expectations. Possible reasons for the result among middle-aged people could be that this group normally is engaged in a number of social activities, which had become reduced during the pandemic. The decreased social network may in turn generate a feeling of loneliness, which has been described previously [15]. Furthermore, studies have shown that a person’s social situation (i.e., family situation and marital status) can impact on loneliness [15] and satisfaction with life [29]. This may explain why the group with no children living at home had significantly higher odds for experiencing deteriorated satisfaction with life as a whole. Furthermore, Bluestein et al. [11] and Zhang et al. [41] have shown that having a precarious income during the pandemic could lead to worry and anxiety, which may explain why the group with other sources of income than being employed had higher odds for experiencing deteriorated life satisfaction in our study [11,41]. As expected, people living in Stockholm also had slightly increased odds for deteriorated life satisfaction, which may be explained by the larger initial outbreaks of COVID-19 and hence stricter restrictions in this area during the first wave of the pandemic. Thus, our findings are partly in agreement with the studies from Europe and other parts of the world that have been locked-down [5,11,39,41], showing that reduced social activities and increased unemployment are associated with lower life satisfaction. Furthermore, in the study by Benke et al. [8], it was reported that women, older age, a higher educational level, being employed, and cohabiting were associated with higher life satisfaction. In the present study, no significant gender difference regarding changes in life as a whole was seen in the multivariate regression analysis. However, other studies have reported that women tend to be more stressed [36], and respond to social isolation with anxiety and depression [36,45], which may impact life satisfaction negatively. In another Swedish study [17], it was reported that older adults overall perceived a high well-being during the early stage of the pandemic. Those who reported lower well-being were, however, worried about negative health and socioeconomic consequences of the pandemic. As only 5% of the participants in the present study were >70 years old, it was difficult to draw any conclusions about their experiences. The group had increased odds for deteriorated satisfaction, compared with the youngest age group but the increased odds were not significant. This may be due to the relatively low number of participants in this age group. Thus, how older people have experienced changes in life satisfaction during the continuation of the pandemic in Sweden needs to be further assessed. That underlying diseases could lead to worry and distress as well as reduced life satisfaction was expected and have been reported earlier, for example in people with diabetes [3] and Parkinson’s disease [4]. This is fully understandable as COVID-19 could be particularly dangerous for people with chronic diseases [3,4].

Taken together, several factors may influence life satisfaction during a pandemic. The Swedish strategy, with more lenient public health restrictions, seems to have affected people´s life satisfaction negatively to some extent, even though a majority perceived a high level of life satisfaction. To gain a deeper understanding of how peoples´ perceived life satisfaction has changed during the pandemic in Sweden, more qualitative studies are needed.

### Strengths and Limitations

A strength of the present study was the relatively large sample size, and that self-reported life satisfaction was assessed with a common and validated rating scale comprising many important life domains (LiSat-11). However, other important psychosocial factors that may influence a person’s life satisfaction could also have been assessed, for example work-related stress, burnout, stigma, discrimination, and self-esteem. By recruiting participants through Facebook, a large geographical area in Sweden was covered, where the outbreak of COVID-19 had varied substantially. However, a limitation of this study was that our online survey attracted a selected group of social media users, mostly middle-aged women, well-educated and married/cohabiting, which may not entirely reflect the general population. Thus, there is a possibility that perceived life satisfaction may have been overestimated; if other groups of people had been included (for example immigrants, people with serious diseases, and younger or even older people), perceived life satisfaction may have been reduced. In our sample, however, the middle-aged group had the highest odds for deteriorated life satisfaction. Regarding the recruitment of participants, previous studies have also used online surveys to investigate how the restrictions and home confinements have influenced peoples’ life situation [5,20]. Facebook could be a useful tool when recruiting participants for health research, because of the short recruitment periods, low costs, and improved participation among young people [49]. However, one drawback is that younger people [50] and women tend to be overrepresented in studies of Facebook recruitment [14,23,49], which is partly in line with the present study. Despite this selection bias, the between-group comparisons could, however, still provide a relevant picture of the differences in perceived changes between the demographic groups.

As this was an explorative study, no sample size calculation was performed. Due to the cross-sectional design, we did not have any pre-pandemic ratings of life satisfaction to compare with. Instead, we asked participants to directly report whether they perceived a change or not in life satisfaction during the past year, and whether they considered the change to be related to the pandemic. However, based on the perspective of implicit theory of change [51], there is a risk that retrospective judgments are biased, and that it is difficult for participants to recall how their actual life situations were back in time. Hence, given our study design and that a selected group of social media users were included in this study, the descriptive results cannot be generalized to the entire population in Sweden.

## 5. Conclusions

A majority of the participants in the present study perceived a high level of life satisfaction during the restrictions of COVID-19 in Sweden, with the exception of contact with friends and sexual life. An equal share, approximately one-third, reported life as a whole as either deteriorated or improved; but a majority of those that perceived a deterioration expressed that it was due to the pandemic. Having no children living at home, being middle aged, living in Stockholm, having other sources of income than being employed, and having a chronic disease were associated with significantly higher odds for experiencing deteriorated satisfaction with life as a whole. Our findings indicate that the Swedish strategy might have contributed to the high proportion of satisfied people. Those who perceived a deterioration in life satisfaction may, however, need attention from Swedish Welfare Authorities.

## Figures and Tables

**Table 1:** Sociodemographic characteristics of the study sample n = 1082.

Variable	Values
**Gender**	
Female, % (n)	82 (882)
Male, % (n)	18 (192)
**Age**	
Mean (SD)	48 (12.2)
**Age groups, % (n)**	
<35	15 (158)
35–49	41 (426)
50–69	40 (421)
70+	5 (47)
**Marital status**	
Single, % (n)	19 (202)
Married/cohabiting, % (n)	74 (852)
Partner: not cohabiting, % (n)	7 (74)
**Family situation**	
Children living at home full time, % (n)	40 (427)
Children living at home part time, % (n)	6 (62)
No children living at home, % (n)	55 (589)
**Geographical area**	
Stockholm, % (n)	22 (240)
Gothenburg, % (n)	15 (159)
Scania, % (n)	43 (466)
Other parts in Sweden, % (n)	20 (213)
**Residential communities**	
Village, % (n)	31 (335)
Town, % (n)	31 (332)
City, % (n)	38 (409)
**Country of birth**	
Sweden, % (n)	91 (980)
Other, % (n)	9 (96)
**Education**	
Upper secondary education, % (n)	15 (165)
Tertiary education, % (n)	85 (913)
**Occupation**	
Employed, % (n)	79 (875)
Other sources of income, % (n)	21 (221)
**Chronic disease**	
Yes, % (n)	26 (280)
No, % (n)	74 (796)

**Table 2:** LiSat-11 scores presented as median (Q1–Q3) and proportion satisfied.

Items	Median (Q1–Q3)	Proportion Satisfied * % (n)
Life as whole (n = 1080)	5 (4–6)	69 (750)
Vocational situation (n = 1070)	5 (4–5)	56 (600)
Financial situation (n = 1081)	5 (4–5)	70 (751)
Leisure (n = 1075)	5 (4–6)	62 (669)
Contact with friends (n = 1078)	4 (4–5)	43 (465)
Sexual life (n = 1065)	4 (3–5)	35 (370)
Activities of daily living (n = 1077)	6 (6–6)	97 (1046)
Family life (n = 1055)	5 (4–6)	73 (768)
Partnership/relationship (n = 926)	5 (4–6)	71 (655)
Physical health (n = 1077)	5 (4–5)	65 (696)
Psychological health (n = 1079)	5 (4–5)	64 (689)

* Satisfied defined as score 5 or 6 on the 1–6 scale; Q1 and Q3 = quartile 1 and 3.

**Table 3:** Reported change in life satisfaction with different aspects of life (LiSat-11) compared to the same period last year, and whether perceived change was experienced due to the COVID-19 restrictions (n = 1076).

Items	Reported Changes in Life Satisfaction (i.e., Deteriorated (-); Unchanged (0); or Improved (+))			If Change: Considered to Be Related to the Pandemic? (Yes or Partly)		*p* *
		%	(n)	%	(n)	
Life as whole	-	28	301	95	285	<0.001
	0	43	463	-	-	
	+	29	312	50	155	
Vocational situation	-	24	259	87	225	<0.001
	0	51	546	-	-	
	+	25	264	46	121	
Financial situation	-	11	121	68	82	<0.001
	0	69	744	-	-	
	+	20	210	32	68	
Leisure	-	33	359	96	343	<0.001
	0	43	458	-	-	
	+	24	258	67	174	
Contact with friends	-	52	560	98	550	<0.001
	0	42	449	-	-	
	+	6	66	65	42	
Sexual life	-	12	123	35	43	0.766
	0	82	873	-	-	
	+	7	73	33	24	
Activities of daily living	-	3	28	79	22	<0.001
	0	94	1004	-	-	
	+	4	42	36	15	
Family life	-	13	144	88	127	<0.001
	0	64	690	-	-	
	+	17	185	68	126	
Partnership/relationship	-	7	70	53	37	0.084
	0	64	682	-	-	
	+	13	138	65	90	
Physical health	-	22	237	71	169	0.001
	0	52	561	-	-	
	+	26	277	58	160	
Psychological health	-	21	226	90	204	<0.001
	0	65	698	-	-	
	+	14	150	54	81	

* Chi square test comparison of proportion of participants attributing the change to the pandemic.

**Table 4:** Proportion of participants reporting changes in satisfaction with life as a whole.

Variable	Deteriorated, % (n)	Unchanged, % (n)	Improved, % (n)	*p*
**Gender**				0.028
Women	29 (255)	41 (361)	30 (259)	
Men	22 (43)	52 (99)	26 (50)	
**Age groups**				<0.001
<35	24 (38)	32 (50)	44 (68)	
35–49	29 (123)	40 (168)	31 (132)	
50–69	26 (109)	50 (209)	24 (100)	
70+	49 (23)	45 (21)	6 (3)	
**Marital status**				0.062
Single	34 (69)	35 (70)	31 (62)	
Married/cohabiting	27 (213)	45 (359)	28 (224)	
Partner: not cohabiting	23 (17)	43 (32)	34 (25)	
**Family situation**				0.006
Children living at home full time	24 (100)	42 (179)	34 (145)	
Children living at home part time	27 (17)	37 (23)	36 (22)	
No children living at home	31 (182)	44 (259)	25 (144)	
**Geographical area**				0.005
Stockholm	36 (86)	34 (80)	30 (72)	
Gothenburg	28 (44)	45 (71)	28 (44)	
Scania	27 (124)	44 (203)	30 (138)	
Other parts in Sweden	22 (45)	52 (108)	27 (56)	
**Residential communities**				0.007
Village	24 (79)	45 (150)	31 (103)	
Town	26 (84)	48 (158)	26 (87)	
City	33 (136)	37 (151)	30 (121)	
**Country of birth**				0.416
Sweden	27 (266)	43 (423)	29 (285)	
Other	34 (32)	40 (38)	26 (25)	
**Education**				0.118
Upper secondary education	26 (43)	50 (82)	24 (39)	
Tertiary education	28 (256)	42 (378)	30 (272)	
**Occupation**				0.001
Employed	25 (214)	44 (376)	30 (258)	
Other sources of income	38 (83)	38 (84)	24 (53)	
**Chronic disease**				0.003
Yes	36 (99)	40 (112)	24 (67)	
No	25 (199)	44 (349)	31 (243)	

**Table 5:** Odds ratio for deteriorated and improved satisfaction with life as a whole, OR (95% CI).

Variable	Deteriorated Satisfaction	Improved Satisfaction
	OR (95% CI)	OR (95% CI)
**Gender**		
Women (n = 843)	1 (ref)	1 (ref)
Men (n = 183)	0.74 (0.50–1.09)	0.88 (0.61–1.28)
**Age groups**		
<35 (n = 154)	1 (ref)	1 (ref)
35–49 (n = 416)	1.75 (1.08–2.85)	0.43 (0.28–0.66)
50–69 (n = 410)	1.01 (0.64–1.60)	0.39 (0.26–0.59)
70+ (n = 46)	1.64 (0.7–3.49)	0.11 (0.33–0.40)
**Family situation**		
Children living at home full time (n = 409)	1 (ref)	1 (ref)
Children living at home part time (n = 60)	1.25 (0.66–2.39)	1.18 (0.67- 2.10)
No children living at home (n = 557)	1.83 (1.28–2.62)	0.63 (0.45–0.89)
**Geographical area**		
Scania (n = 447)	1 (ref)	1 (ref)
Stockholm (n= 231)	1.55 (1.00–2.40)	1.07 (0.69–1.67)
Gothenburg (n = 152)	0.89 (0.56–1.43)	0.93 (0.59–1.47)
Other parts in Sweden (n = 196)	0.73 (0.48–1.11)	1.03 (0.69–1.52)
**Residential communities**		
Village (n = 316)	1 (ref)	1 (ref)
Town (n = 319)	1.07 (0.73–1.56)	0.82 (0.57–1.18)
City (n = 391)	1.21 (0.79–1.85)	0.91 (0.60–1.39)
**Occupation**		
Employed (n = 818)	1 (ref)	1 (ref)
Other sources of income (n = 208)	1.65 (1.13–2.06)	0.90 (0.61–1.34)
**Chronic disease**		
No (n = 760)	1 (ref)	1 (ref)
Yes (n = 266)	1.50 (1.10–2.41)	0.80 (0.57–1.11)

## Data Availability

Data are available only upon request to the authors, according to the ethical approval from the Swedish Ethical Authority.
